# Homochiral Supramolecular Thin Film from Self-Assembly of Achiral Triarylamine Molecules by Circularly Polarized Light

**DOI:** 10.3390/molecules25020402

**Published:** 2020-01-18

**Authors:** Changjun Park, Jinhee Lee, Taehyoung Kim, Jaechang Lim, Jeyoung Park, Woo Youn Kim, Sang Youl Kim

**Affiliations:** 1 Department of Chemistry, Korea Advanced Institute of Science and Technology (KAIST), Daejeon 34141, Korea; pcjhenry@kaist.ac.kr (C.P.); solwal@kaist.ac.kr (J.L.); thkim93@kaist.ac.kr (T.K.); ljchang94@kaist.ac.kr (J.L.); wooyoun@kaist.ac.kr (W.Y.K.); 2 Research Center for Bio-based Chemistry, Korea Research Institute of Chemical Technology (KRICT), Ulsan 44429, Korea; jypark@krict.re.kr; 3 Advanced Materials and Chemical Engineering, University of Science and Technology (UST), Daejeon 34113, Korea

**Keywords:** homochirality, self-assembly, triarylamine, circularly polarized light, helical systems, spontaneous symmetry breaking

## Abstract

Here, we report the formation of homochiral supramolecular thin film from achiral molecules, by using circularly polarized light (CPL) only as a chiral source, on the condition that irradiation of CPL does not induce a photochemical change of the achiral molecules. Thin films of self-assembled structures consisting of chiral supramolecular fibrils was obtained from the triarylamine derivatives through evaporation of the self-assembled triarylamine solution. The homochiral supramolecular helices with the desired handedness was achieved by irradiation of circularly polarized visible light during the self-assembly process, and the chiral stability of supramolecular self-assembled product was achieved by photopolymerization of the diacetylene moieties at side chains of the building blocks, with irradiation of circularly polarized ultraviolet light. This work provides a novel methodology for the generation of homochiral supramolecular thin film from the corresponding achiral molecules.

## 1. Introduction

Chirality is one of the most universal and intriguing phenomena, owing to its presence in nature at all length scales, from atomic to galactic levels [1,2]. In particular, supramolecular chirality has received considerable attention as it is strongly related to the homochiral systems in living organisms, such as DNA, RNA, and proteins [3]. Constructing supramolecular systems with chirality also attracts significant interests due to their potential applications in chiral recognition, optoelectronics and asymmetric catalysts [4]. Most chiral supramolecular structures are generated from self-assembly of chiral monomers or addition of chiral dopants into achiral molecules [5]. Nevertheless, supramolecular chirality is also generated from spontaneous symmetry breaking of supramolecular assemblies that are only composed of achiral molecules [6]. This process is especially important since an understanding of spontaneous mirror symmetry breaking mechanism can provide knowledge necessary to unveil the origins of homochirality in nature [7].

The spontaneous symmetry breaking of self-assembled structures based on achiral molecules can be achieved by purely physical fields, such as circularly polarized light (CPL) irradiation [8,9,10,11,12,13,14,15,16], hydrodynamic shear force by stirring [6,7,17,18] or the combination of light, magnetic, hydrodynamic or thermal stimuli [19,20]. Among them, CPL is considered to be a true chiral entity and has been regarded as the origin of homochirality in nature [21,22]. Photochromic molecules undergo isomerization by irradiation of CPL and supramolecular chirality is exhibited through an alignment of molecules with the same handedness as that of CPL [8,9,10]. Light-initiated self-assembly systems have also been employed for supramolecular chirality by using CPL to initiate and facilitate the stacking of building blocks through conformational change, and to simultaneously control the handedness of self-assembled structures [11,12,13]. In addition, the formation of chiral structures can be achieved from achiral one-dimensional supramolecular assemblies through a helical polymerization that induces the rotational direction [14,15,16]. However, generation of supramolecular chirality by irradiation of CPL is restricted to the case that light affects monomers, causing the change of properties, and the investigation of the pure effect of CPL as a chiral source at the generation of supramolecular chirality is hindered.

Herein, we report the spontaneous symmetry breaking of self-assembled structures based on achiral molecules by using CPL only as a chiral source. Consequently, irradiation of CPL does not induce the photochemical change of achiral molecules (supramolecular synthons), to precisely investigate the effects of CPL on the spontaneous symmetry breaking. A triarylamine (TAA) moiety is used as a building block of the supramolecular assemblies to form one-dimensional self-assembled structures, through π–π interaction [23]. Self-assembly of TAA derivatives has been investigated in chlorinated solvents by irradiation of light, causing the formation of triarylammonium radicals with more planar structures, compared to neutral TAA structures and promoting stacking through charge transfer [24,25,26]. In our previous work, we achieved the formation of helical self-assembled structures composed of achiral TAA molecules in the chlorinated solvent and simultaneous control of the rotational direction by using CPL, with the desired handedness [12]. Nevertheless, TAA derivatives have been also known to self-assemble by heating and the subsequent cooling process, mainly through π–π interaction [27,28,29]. Furthermore, the supramolecular chirality can be induced by the TAA building blocks with chiral side chains [30,31,32]. Compared with the reported methodologies, the supramolecular chirality is generated from achiral TAA-containing molecules in non-chlorinated solvents by irradiation of CPL, without generating radicals. To promote the self-assembly of the TAA derivatives through the enhanced noncovalent interactions, a stilbene moiety as an aryl group and long alkyl group as a side chain are incorporated. The amide group is also incorporated to induce noncovalent interactions via hydrogen bonding for helical supramolecular assemblies [33,34,35]. In addition, the solution casting method is applied to make a self-assembled supramolecular thin film consisting of the TAA derivatives. As a solvent, benzene is used for dissolution of the TAA building blocks at an increased temperature and formation of the self-assembled supramolecular structure was induced by cooling, and then the solvent was evaporated to obtain self-assembled supramolecular thin film. Moreover, locking of the supramolecular chirality is achieved through topochemical photopolymerization of diacetylene (DA) moieties [36,37], incorporated at the side chains of the TAA building blocks, through irradiation of circularly polarized ultraviolet light (CPUL), which give rise to a remarkable stability of the polymerized chiral supramolecular structure. To the best of our knowledge, this is the first work of the homochiral supramolecular systems based on achiral building blocks where the CPL is used only as a chiral source without a photochemical change of the building blocks.

## 2. Results and Discussion

### 2.1. Synthesis and Self-Assembly

**Scheme 1:**
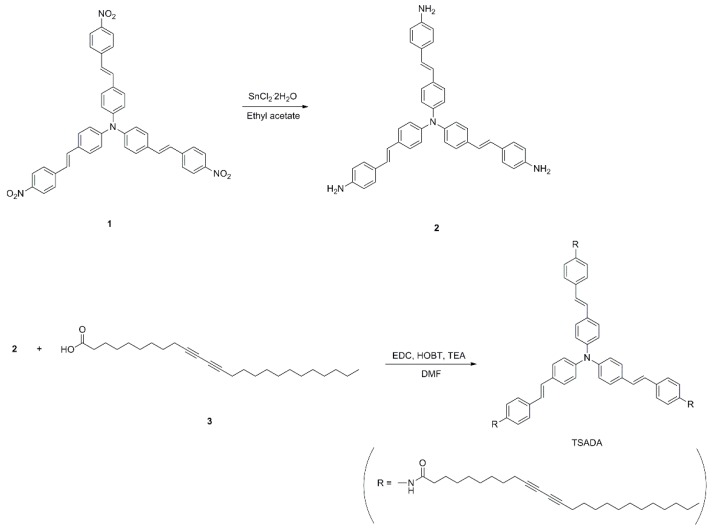
Synthesis of TSADA from a nitro-substituted TAA compound **1** through reduction and subsequent amide coupling reaction.

The synthetic routes of achiral compound containing tris(stilbene)amine (TSA) and diacetylene (DA) moieties are shown in Scheme 1. We named the achiral compound as TSADA, due to the presence of the TSA moiety at the core and DA moieties at the side chains of the molecule. Reduction of a nitro-substituted TAA compound **1** produced an amine-substituted TAA compound **2**. TSADA was synthesized by amide coupling reaction between **2** and a carboxylic acid-substituted DA compound **3**.

**Figure 1:**
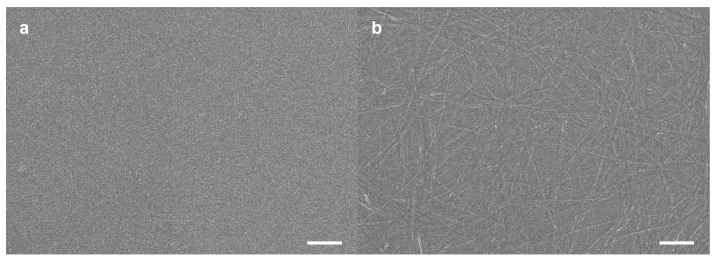
FE–SEM images of the aggregates of TSADA formed in benzene solution (1 mg/mL). Samples were prepared by spin coating of the benzene solution of TSADA (**a**) without heating and (**b**) after heating and subsequent cooling. Scale bar, 500 nm.

TSADA was dissolved well in tetrahydrofuran (THF) at room temperature and the ^1^H nuclear magnetic resonance (NMR) spectrum of TSADA in deuterated THF (THF-*d*_8_) showed distinct signals of the aromatic and amide protons. However, the signals of aromatic and amide protons disappeared at the ^1^H NMR spectrum of TSADA in deuterated benzene (benzene-*d*_6_) (Figure S1a). In the case of the benzene-*d*_6_ solution, temperature-dependent ^1^H NMR spectral changes were monitored to check the effect of the heating and cooling process. The signals appeared after heating of the solution to 70 °C (Figure S1b), and cooling of the solution to room temperature gave rise to the disappearance of the signals again (Figure S1c), indicating that the aggregates of TSADA were formed in benzene at room temperature. The morphological changes of the aggregates were visualized by field-emission scanning electron microscopy (FE–SEM). Although the FE-SEM image revealed no obvious structures before heating the benzene solution of TSADA, one-dimensional self-assembled structures of TSADA were observed when the heated benzene solution was cooled to room temperature (Figure 1). The results indicated that the disappearance of signals corresponding to the aromatic and amide protons at the ^1^H NMR spectrum of TSADA in benzene-*d*_6_ after heating and subsequent cooling originated from the stacking of TSADA through π–π interaction and amide hydrogen bonding, which is consistent with the literature on self-assembly of TAA [12,24,26]. FE–SEM image of self-assembled structures showed the formation of nanofibers that were several micrometers in length and had an approximate width of 30 nm on average. Transmission electron microscopy (TEM) images of the self-assembled structures showed consistent observations (Figure S2). The absorption band of TAA core in TSADA became broader due to the formation of aggregates (Figure S3). In addition, an attenuated total reflection infrared (ATR–IR) spectrum of TSADA exhibited the presence of amide hydrogen bonding between the stacked TSADA moieties (Figure S4).

**Figure 2:**
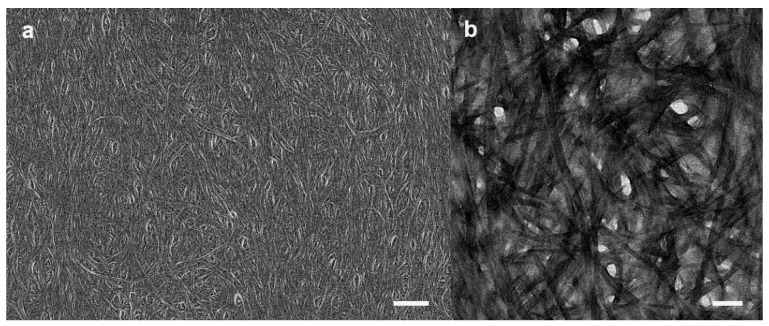
Microscopic images of the supramolecular thin film consisting of the self-assembled structures of TSADA from benzene solution (1 mg/mL) obtained by solution casting. (**a**) FE–SEM image of the supramolecular thin film. Scale bar, 1 μm. (**b**) TEM image of the supramolecular thin film. Scale bar, 200 nm.

To produce the supramolecular thin film of TSADA, the benzene solution of TSADA was casted and the morphology of the self-assembled thin film was visualized by FE–SEM. Solution casting of TSADA in benzene produced network structures consisting of fibrillar aggregates (Figure 2a). The fibrillar bundle was composed of nanofibers that were approximately 30 nm wide on average, and the bundles of the one-dimensional self-assembled structures of TSADA constituted the supramolecular thin film. The consistent image of the supramolecular thin film was observed with TEM (Figure 2b). X-ray diffraction (XRD) study of the supramolecular thin film was carried out to analyze the self-assembled structures of TSADA. The wide-angle XRD pattern showed peaks at 2*θ* = 21.96° and 18.95° referring to spacing (d) of 4.02 Å and 4.66 Å, respectively, which were ascribed to the intermolecular distance between the stacked TSADA building blocks (Figure S5a). The small-angle XRD pattern revealed a peak at 2*θ* = 2.27° referring to d = 3.89 nm, which was ascribed to the chain length of the TSADA moieties (Figure S5b). The self-assembled structure of TSADA was computationally simulated and the computational result suggested that the predicted geometry of the self-assembled structures of TSADA showed helical stacking due to the hydrogen bonding of the amides, which was consistent with the literature (Figure S6) [33,34,35].

### 2.2. Formation of Homochiral Supramolecular Thin Film by CPL

**Figure 3:**
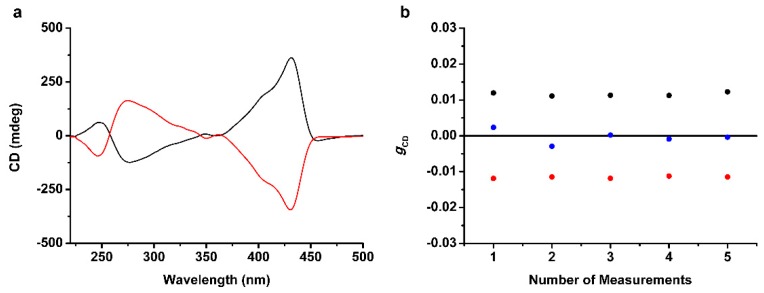
Supramolecular chirality of the thin film of TSADA obtained by self-assembly and solution casting of the benzene solution (1 mg/mL). (**a**) CD spectra with control of the rotational direction of the self-assembled structures by irradiation of *l*-CPL (circularly polarized light) (black line) or *r*-CPL (red line) during self-assembly and solution casting. (**b**) The *g*_CD_ values of the supramolecular thin film of TSADA without control (blue dots) or with control of the rotational direction by using *l*-CPL (black dots) or *r*-CPL (red dots). The measurements were performed at the center and four edges of the substrates to cover all areas of the supramolecular thin film.

**Scheme 2:**
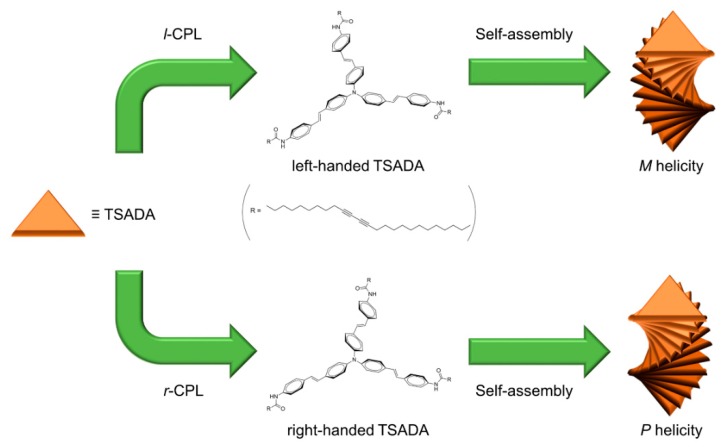
Schematic illustration of the generation of homochiral supramolecular assemblies by irradiation of circularly polarized light (CPL). Irradiation of CPL gives rise to enantiomerization of TSADA, depending on the rotational direction of CPL and generates self-assembled structures with controlled handedness.

Irradiation of *l*-CPL during the self-assembly and solution casting of the benzene solution of TSADA produced intense circular dichroism (CD) activity with positive maxima at 431 and 244 nm, and a negative maximum at 278 nm (Figure 3a, black line). Irradiation of *r*-CPL also exhibited same results, showing the opposite Cotton effect (Figure 3a, red line). These observations clearly indicated that the handedness of self-assembled structures composing supramolecular thin film could be selectively generated by irradiation of CPL with the same handedness. In addition, the measurements of the anisotropy factor *g*_CD_—the ratio of molar CD to molar extinction coefficient—was performed to quantify the intensity of the supramolecular chirality (Figure 3b). In the case of the formation of supramolecular thin film without irradiation of CPL, the rotational direction of helical assemblies was uncontrolled. As a result, the average value of the *g*_CD_ at λ = 431 nm, with the measurements at five different positions of single supramolecular thin film, came close to zero. Although chiral domains with weak absolute *g*_CD_ values were observed due to the heterogeneous condition of solid thin film rather than homogeneous solution, the result suggested that supramolecular assemblies with *M* helicity and *P* helicity existed with equal probabilities. Compared with the thin film prepared without CPL, intense average absolute value of the *g*_CD_ at λ = 431 nm (estimated to be 0.0115) was obtained for the supramolecular thin film with control of the handedness by CPL. Moreover, the measurement of *g*_CD_ values at different positions exhibited consistent observations, suggesting that a homochiral supramolecular structure was produced, which was consistent with the literature [38]. It seems that the electromagnetic field of CPL with the presence of angular momentum generated preferential enantiomeric form of TAA derivatives. As shown in Figure S3, the TAA core of TSADA absorbs the visible light with a range of wavelength from 400 to 450 nm. Accordingly, TSADA reached to excited states by irradiation of CPL without radical generation and the π electrons with the dipole moment was affected by the angular momentum of CPL, resulting in an enantiomerization of TSADA. One enantiomeric form of TSADA having a propeller-like structure of triaryl rings (left-or right-handed) served as a chiral molecule for supramolecular assemblies, by following the “Sergeants and Soldiers” principle [39,40]. During the self-assembly and solution casting, stacking of TSADA derivatives only with the same enantiomeric form would be achieved. Consequently, the desired handedness of self-assembled structures would be generated by controlling the rotational direction of CPL. Schematic illustration of the generation of homochiral supramolecular assemblies by irradiation of CPL, based on the description above, is shown in Scheme 2. The experiments were repeated to confirm the reliability of chirality control of the supramolecular structure by CPL and the consistent results were obtained by using CPL with the same rotational direction (Figure S7). We also noted that films composed of chiral supramolecular assemblies exhibited consistent CD activity with rotation and inversion of the homochiral thin films, indicating that the CD activity mainly originated from the controlled helicity of the supramolecular assemblies (Figure S8) [13,14]. Furthermore, linear dichroism (LD) measurements against the rotation angle in the film plane were carried out to investigate the contribution of macroscopic anisotropy to the CD activity [41,42,43]. The contribution of the LD signal to the CD signal was estimated to be 0.06%, suggesting that the LD contribution to the CD signals was negligible (Figure S9).

The exposure of the homochiral supramolecular structure to ambient light caused the disappearance of CD activity due to the racemization of the supramolecular structures (Figure S10a). In addition, time-dependent changes of CD activity were analyzed during irradiation of *r*-CPL on the homochiral supramolecular thin film of TSADA, which was prepared by irradiation of *l*-CPL during the self-assembly and solution casting (Figure S11a). The initial CD activity induced by *l*-CPL disappeared and then the CD signal of the opposite sign increased, revealing that the inversion of supramolecular chirality occurred by irradiation of CPL with the opposite handedness. The maximal intensity was obtained after 5 h of irradiation but the intensity was relatively low compared to the initial CD activity with the opposite sign, suggesting that the rotational direction was not fully inversed. Moreover, the CD activity hardly changed, despite further additional irradiation, regardless of the handedness of CPL. Irradiation of *l*-CPL followed by irradiation of *r*-CPL on the homochiral supramolecular thin film of TSADA, which was prepared by irradiation of *r*-CPL during the self-assembly and solution casting, showed an identical result, except they showed an opposite Cotton effect (Figure S11b). These observations suggest that the self-assembled structures of TSADA were locked during the inversion of the rotational direction, probably because of the photopolymerization of DA moieties. Disappearance of the CD signal at 244 nm, corresponding to the absorbance of DA moieties supports the photopolymerization. To investigate the photopolymerization of DA moieties, Fourier transform Raman (FT–Raman) spectral changes of the supramolecular thin film were monitored. Although the formation of the chiral supramolecular thin film through the self-assembly and solution casting was performed without irradiation of ultraviolet light, weak signals appeared at 1507 and 2110 cm^−1^ (Figure S12b). These signals correspond to *v*(C = C) and *v*(C ≡ C) of the polymerized DA, respectively, suggesting that topochemical polymerization of DA moieties was already initiated. As a comparison, the aggregates of TSADA without the heating and cooling process exhibited no signals at 1507 and 2110 cm^−1^ (Figure S12a). These results suggest that a slight topochemical polymerization of the DA moieties had occurred during the dissociation of the aggregates by heating. In addition, a remarkable increase of the peaks referring to *v*(C = C) and *v*(C ≡ C) of the polymerized DA was observed by continuous irradiation of visible light to the chiral supramolecular thin film (Figure S12c) [44].

### 2.3. Chiral Stability of the Photopolymerized Homochiral Thin Film

As described above, the supramolecular chirality of the homochiral thin film was affected by non-polarized light or CPL with the opposite handedness. However, the maintenance of the CD activity against irradiation of CPL regardless of the rotational direction was observed when the DA moieties of TSADA were polymerized. Consequently, the polymerization of DA moieties of the homochiral supramolecular thin film was performed to induce the chiral stability. The locking of self-assembled structures of the chiral supramolecular thin film was achieved by irradiation of ultraviolet light to photopolymerize the DA moieties. A FT-Raman spectrum of the polymerized thin film exhibited the distinct peaks at 1507 and 2110 cm^−1^ corresponding to *v*(C = C) and *v*(C ≡ C) of the polymerized DA, respectively (Figure S12d). We also visualized the morphology of the polymerized thin film by FE–SEM and TEM (Figure S13). The obtained images revealed the network structures consisting of densely aggregated fibrils, similar to the images of non-polymerized sample, as shown in Figure 2. In addition, XRD measurements of the polymerized thin film showed consistent patterns when compared to the XRD patterns of the non-polymerized sample (Figure S14). These observations indicate that the one-dimensional self-assembled structures generated from the stacking of TSADA were retained during the polymerization.

**Figure 4:**
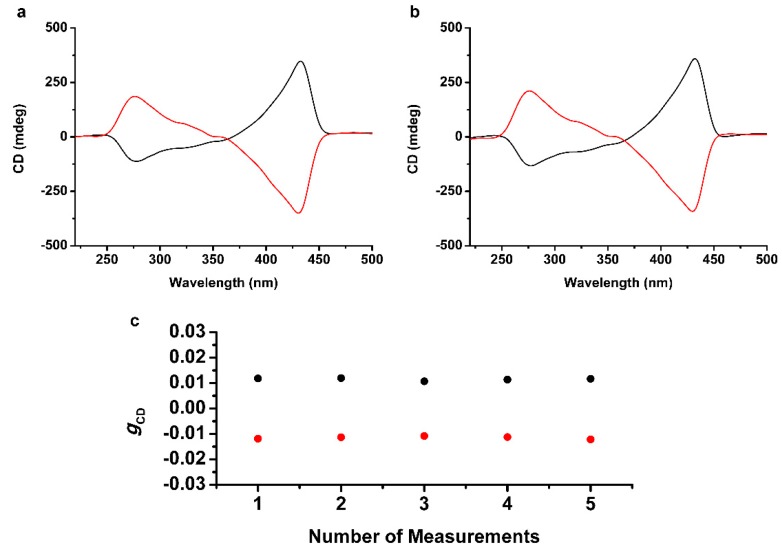
Locking of the induced handedness by circularly polarized ultraviolet light (CPUL). (**a**) CD spectra of polymerized homochiral thin film of TSADA, prepared by irradiation of CPUL with the same handedness of the self-assembled structures. Irradiation of *l*-CPUL was performed to the homochiral supramolecular thin film prepared by *l*-CPL (black line) and irradiation of *r*-CPUL was performed to the homochiral supramolecular thin film prepared by *r*-CPL (red line). (**b**) Maintenance of the CD activity of the polymerized homochiral thin film of TSADA against irradiation of CPL with the opposite handedness. CD spectra were recorded after irradiation of *r*-CPL to the polymerized homochiral thin film prepared by *l*-CPL, *l*-CPUL (black line), and *l*-CPL to the polymerized homochiral thin film prepared by *r*-CPL and *r*-CPUL (red line). (**c**) The *g*_CD_ values of the polymerized homochiral thin film of TSADA prepared by using *l*-CPL and *l*-CPUL (black dots) or *r*-CPL and *r*-CPUL (red dots). The measurements were performed at the center and the four edges of the substrates to cover all areas of the thin film.

The exposure of the homochiral supramolecular thin film to non-polarized ultraviolet light also caused the racemization and exhibited no CD activity (Figure S10b). Consequently, the homochiral supramolecular thin film was photopolymerized to maintain the induced handedness by irradiation of CPUL. Irradiation of *l*-CPUL to the supramolecular thin film of TSADA, prepared by irradiation of *l*-CPL during the self-assembly and solution casting, retained the initial CD activity with the disappearance of the peak at 244 nm (Figure 4a, black line). The result was identical when using *r*-CPL and *r*-CPUL, except for an opposite Cotton effect (Figure 4a, red line). Moreover, the polymerized homochiral thin film retained the CD activity against the irradiation of CPL, in the opposite rotational direction, for 6 h, indicating that the chiral stability was achieved (Figure 4b). The measurements of the *g*_CD_ value of the polymerized homochiral thin film at different positions showed that the homochirality of the supramolecular assemblies was maintained (Figure 4c).

## 3. Materials and Methods

### 3.1. Materials and Characterization

Tin (II) chloride dihydrate (98%), 1-hydroxybenzotriazole hydrate (HOBT) (97%), and triethylamine (TEA) (99.5%) were purchased from Sigma-Aldrich (St. Louis, MO, USA). 1-(3-Dimethylaminopropyl)-3-ethylcarbodiimde (EDC) (98%) was purchased from Tokyo Chemical Industry Co., Ltd. (Tokyo, Japan). 10,12-Pentacosadiynoic acid (compound **3**) (98%) was purchased from Alfa Aesar (Heysham, UK). Ethyl acetate (99.5%), *N*,*N*-dimethylformamide (DMF) (99.5%) and benzene (99.5%) were purchased from Junsei Chemical Co., Ltd. (Tokyo, Japan). All chemicals were used as received. A nitro-substituted TAA compound **1** was synthesized according to the literature procedure [45].

^1^H and ^13^C-NMR spectra of synthesized compounds were recorded on a Bruker Science Avance 400 MHz NMR spectrometer. Elemental analysis was performed on a Thermo Scientific FLASH 2000 series elemental analyzer. High-resolution mass spectroscopy (HR-MS) was performed on a Bruker Daltonics micrOTOF-Q II mass spectrometer using the electrospray ionization method. Matrix-assisted laser desorption ionization time-of-flight (MALDI-TOF) mass spectrum was recorded on a Bruker Daltonics Autoflex III mass spectrometer in the reflection mode using Nd: YAG laser (λ = 355 nm) and a sample was prepared with α-cyano-4-hydroxycinnamic acid (CHCA) as a matrix. FE–SEM was performed on a Hitachi SU8230 SEM. Samples were prepared on a silicon wafer by spin coating or solution casting of the benzene solution of TSADA (1 mg/mL). The samples were coated with Pt before imaging. TEM was performed on an FEI company Tecnai F20 TEM. Samples were prepared on a 200-mesh carbon film by dropping benzene solution of TSADA (1 mg/mL) and through evaporation of the solvent. The samples were stained by exposing ruthenium tetroxide vapor before imaging. UV-vis spectroscopy was conducted using a Shimadzu UV-2600 spectrophotometer. ATR-IR spectroscopy was performed on a Thermo Scientific Nicolet iN10 MX Fourier transform infrared spectrometer. A sample was prepared by solution casting of benzene solution of TSADA (1 mg/mL) on a quartz substrate. XRD patterns were conducted using a Rigaku D/MAX-2500 X-ray diffractometer with a scan speed of 2° per minute and sampling width of 0.01°. Cu *K*_α_ (λ = 0.154 nm) was used as a light source. Samples were prepared by solution casting of benzene solution of TSADA (1 mg/mL) on a silicon wafer and a quartz substrate for wide-angle XRD and small-angle XRD, respectively. CD and LD spectra were obtained on a Jasco Inc., J-815 spectropolarimeter. Samples were prepared by solution casting of benzene solution of TSADA (1 mg/mL) on a quartz substrate and measurement was conducted at room temperature using a scan speed of 200 nm/min and sampling width of 1 nm. The *g*_CD_ value was estimated as *g*_CD_ = [CD intensity/32980]/absorbance at 431 nm, which is the wavelength that exhibits a maximum value of CD intensity. To cover all areas of the supramolecular thin film, the *g*_CD_ values were recorded at the center and the four edges of the substrate. Angle-dependent CD and LD measurements by rotating the supramolecular thin film in steps of 10° was performed to estimate the contribution of the LD signal to the CD signal. FT-Raman spectra were recorded on a Horiba LabRAM HR Evolution spectrometer, using laser at a wavelength of 1064 nm. Samples were prepared by solution casting of benzene solution of TSADA (1 mg/mL) on a quartz substrate.

### 3.2. Synthetic Procedures and Methods

#### 3.2.1. Synthesis of an Amine-Substituted TAA Compound 2

Compound **1** (0.824 g, 1.20 mmol) and tin (II) chloride dihydrate (8.14 g, 36.0 mmol) were dissolved in 150 mL of ethyl acetate. The reaction mixture was refluxed for 12 h. After reaction, the reaction mixture was filtrated through celite. The mixture was basified to pH 11 with aqueous solution of sodium hydroxide and extracted with tetrahydrofuran. The combined organic layer was dried by anhydrous sodium sulfate and condensed in a rotary evaporator. The crude product was precipitated in hexane, and pure **2** as a yellow solid (0.487 g, yield 68.0%) was obtained through filtration. HR-MS, m/e 597.30 for [M + H]^+^ (Calcd.: 596.76). δ_H_ (400 MHz, DMSO-*d*_6_; p.p.m.) 7.44 (6H, *d*, *J* = 8.8 Hz, Ar), 7.26 (6H, *d*, *J* = 8.6 Hz, Ar), 6.98 (6H, *d*, *J* = 8.7 Hz, Ar), 6.96 (3H, *d*, *J* = 16.3 Hz, NC_6_H_4_C*H*), 6.86 (3H, *d*, *J* = 16.3 Hz, NC_6_H_4_CH = C*H*), 6.56 (6H, *d*, *J* = 8.6 Hz, Ar), 5.28 (6H, *s*, N*H_2_*). δ_C_ (100 MHz, DMSO-*d_6_*; p.p.m.) 149.01, 145.76, 133.26, 128.35, 127.89, 127.29, 125.42, 124.20, 122.72, 114.36. Anal. Calcd. for C_42_H_36_N_4_: C 84.53, H 6.08, N 9.39; Found: C 83.33, H 6.16, N 9.18.

#### 3.2.2. Synthesis of TSADA

Compound **2** (0.487 g, 0.816 mmol), **3** (1.38 g, 3.67 mmol), and HOBT (0.772 g, 5.71 mmol) were dissolved in 100 mL of DMF. The reaction mixture was stirred at 0 °C. After 30 min, EDC (1.09 g, 5.71 mmol) and TEA (0.578 g, 5.71 mmol) were added into solution and the reaction mixture was warmed up to room temperature. The reaction mixture was stirred for 12 h. After reaction, the reaction mixture was poured into brine and extracted with THF. The combined organic layer was dried by anhydrous magnesium sulfate and condensed in a rotary evaporator. The crude product was purified by column chromatography on silica gel with THF/hexane 1:1 as an eluent and was recrystallized in ethanol to obtain pure TSADA as an orange solid (0.780 g, yield 57. 4%). MALDI-TOF-MS, *m*/*z* 1667.29 for [M + H]^+^ (Calcd: 1666.50). δ_H_ (400 MHz, THF-*d*_8_; p.p.m.) 9.06 (3H, s, N*H*CO), 7.61 (6H, *d*, *J* = 8.7 Hz, Ar), 7.44 (6H, *d*, *J* = 8.7 Hz, Ar), 7.42 (6H, *d*, *J* = 8.7 Hz, Ar), 7.06 (6H, *d*, *J* = 8.7 Hz, Ar), 7.04 (6H, *s*, C6H4C*H*), 2.29 (6H, *t*, *J* = 7.4 Hz, COC*H_2_*), 2.23 (12H, *t*, *J* = 6.6 Hz, C≡CC*H_2_*), 1.68 (6H, *p*, *J* = 7.0 Hz, COCH_2_C*H_2_*), 1.54-1.44 (12H, *m*, C≡CCH_2_C*H_2_*), 1.44–1.22 (78H, m, COCH_2_CH_2_(C*H_2_*)_4_, (C*H_2_*)_9_CH_3_), 0.88 (9H, *t*, *J* = 6.9 Hz, C*H_3_*). δ_C_ (100 MHz, THF-*d_8_*; p.p.m.) 171.82, 148.10, 140.79, 134.37, 134.04, 128.69, 128.56, 128.03, 127.79, 125.60, 120.46, 78.10, 68.60, 68.38, 68.16, 67.02, 38.37, 33.50, 31.24, 31.22, 31.20, 31.10, 30.93, 30.89, 30.70, 30.60, 30.39, 30.04, 26.96, 26.48, 26.28, 26.08, 24.19, 20.21, 15.07. Anal. Calcd. for C_117_H_156_N_4_O_3_: C 84.32, H 9.44, N 3.36; Found: C 83.88, H 9.43, N 3.62.

#### 3.2.3. Density-Functional-Tight-Binding (DFTB) Simulation

The self-assembled structure of TSADA was computationally simulated by density-functional-tight-binding (DFTB) calculations [46]. Although ab initio calculations usually provide reliable optimized geometry, the huge size of TSADA hampers high-level quantum calculations such as density functional theory (DFT). For instance, TSADA pentamer consists of 1400 atoms and an even simplified pentamer with *n*-pentyl side chains has 665 atoms. Consequently, the geometry of the self-assembled structure of TSADA was obtained from DFTB calculations with dispersion correction by DFT-D3 by considering all atoms without omitting [47,48]. The additional short-range hydrogen–hydrogen repulsion term was also included [49]. DFTB calculations with such corrections provided comparable results to DFT calculations for organic systems with much smaller computational costs, enabling dealing with much larger systems [50]. All calculations were performed using DFTB+ [51]. To prepare the initial geometry of the self-assembled structure, we performed a monomer calculation using DFTB+ with the same conditions as explained above. Then, we stacked the optimized structure of the monomer with an N–N distance of 4.35 Å and a rotation angle of 10° as initial conditions.

#### 3.2.4. Formation of Homochiral Supramolecular Thin Film and Polymerization by Light

Irradiation of light during the self-assembly, solution casting, and polymerization was performed using a Lumatec SUV-DC-P as a light source that had a wavelength range of 280–500 nm and a maximum intensity of 370 nm. To irradiate visible light, a filter with 400 nm of cut-off wavelength was placed in front of the light source. Circularly polarization was performed by placing a linear polarizer and a λ/4 wave-plate in front of the light source. Samples were placed at 5 cm from the lamp and external light was prevented to precisely investigate the effect of CPL.

## 4. Conclusions

In this research, we demonstrated the spontaneous symmetry breaking of the supramolecular assemblies composed of achiral molecules in the condition where incident light does not induce photochemical property change of the building blocks. The handedness of self-assembled structures could be controlled by irradiation of CPL during self-assembly and solution casting and the exposure to CPUL with the same rotational direction permanently locked the induced handedness with remarkable chiral stability. The results not only provide a novel methodology for generation of homochiral supramolecular films for future applications, but also provide a critical clue for the understanding of evolution of the supramolecular chirality through spontaneous mirror symmetry breaking by light.

## Supplementary Materials

The following are available online, Figure S1: Temperature-dependent spectral change in deuterated benzene. Figure S2: TEM images of the aggregates of TSADA in benzene. Figure S3: UV-vis absorbance spectra of TSADA. Figure S4: ATR-IR spectra of the one-dimensional self-assembled structure of TSADA in benzene. Figure S5: XRD patterns of supramolecular assemblies. Figure S6: Estimated structure and supramolecular assembly of TSADA. Figure S7: Repetition of generating homochiral supramolecular structure. Figure S8: Rotation and inversion of homochiral supramolecular thin film. Figure S9: Estimation of the contribution of LD to CD. Figure S10: Racemization of the supramolecular structures under non-polarized light. Figure S11: Time-dependent CD spectral changes of the homochiral supramolecular thin film during irradiation of CPL with opposite rotational direction. Figure S12: Locking of the self-assembled structures by photopolymerization. Figure S13: Microscopic images of the polymerized thin film of TSADA, Figure S14: XRD patterns of polymerized structures.
