# Supplementary material for: Homochiral Supramolecular Thin Film from Self-Assembly of Achiral Triarylamine Molecules by Circularly Polarized Light
Source: Molecules. 2020 Jan 18;25(2):402. doi: 10.3390/molecules25020402 (PMC7024168; doi:10.3390/molecules25020402)
Supplement: Supplementary file 1 [file molecules-25-00402-s001.pdf]

Supplementary Materials

Homochiral Supramolecular Thin Film from Self-Assembly of Achiral Triarylamine Molecules by Circularly Polarized Light

Changjun Park [1], Jinhee Lee [1], Taehyoung Kim [1], Jaechang Lim [1], Jeyoung Park [2,3], Woo Youn Kim [1] and Sang Youl Kim [1,*]

[1] Department of Chemistry, Korea Advanced Institute of Science and Technology (KAIST), Daejeon 34141, Korea.
[2] Research Center for Bio-based Chemistry, Korea Research Institute of Chemical Technology (KRICT), Ulsan 44429, Korea.
[3] Advanced Materials and Chemical Engineering, University of Science and Technology (UST), Daejeon 34113, Korea.
[*] Correspondence: kimsy@kaist.ac.kr; Tel.: (+)82-42-350-2834.

Supplementary Figures

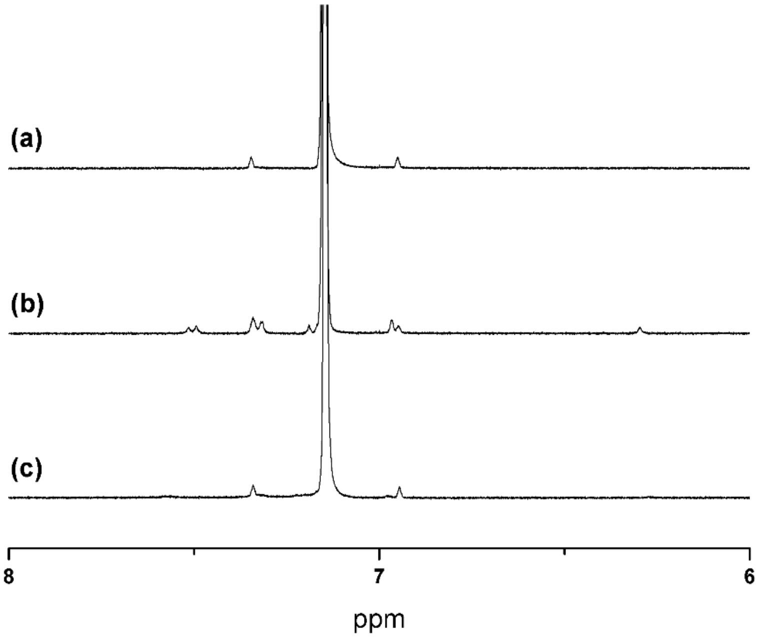

Figure S1. Temperature-dependent spectral change in deuterated benzene. ^{1}H NMR (400 MHz) spectra of TSADA (a) at room temperature before heating, (b) at 70 °C, and (c) at room temperature after heating and subsequent cooling.

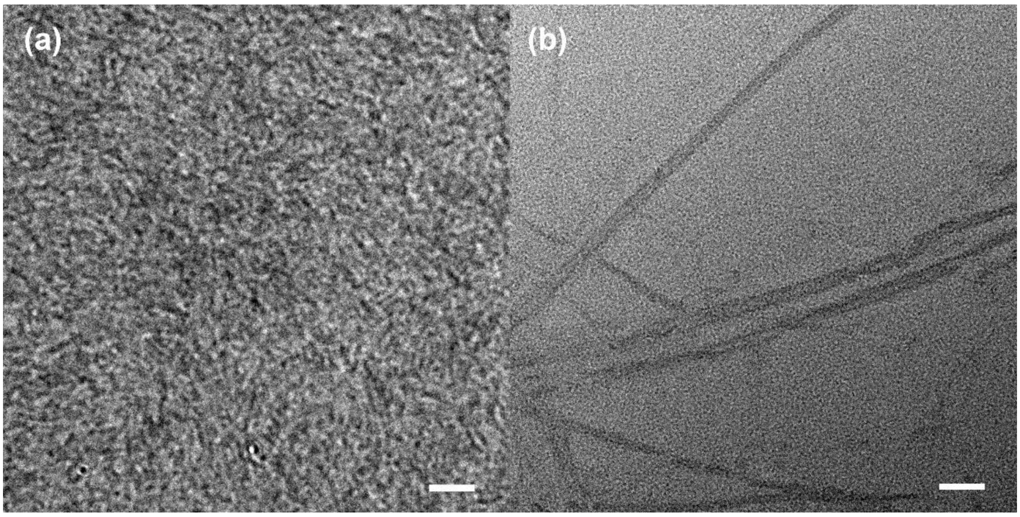

Figure S2. TEM images of the aggregates of TSADA in benzene. (a) The aggregates of TSADA before heating. (b) The aggregates of TSADA after heating and subsequent cooling. Scale bar, 100 nm.

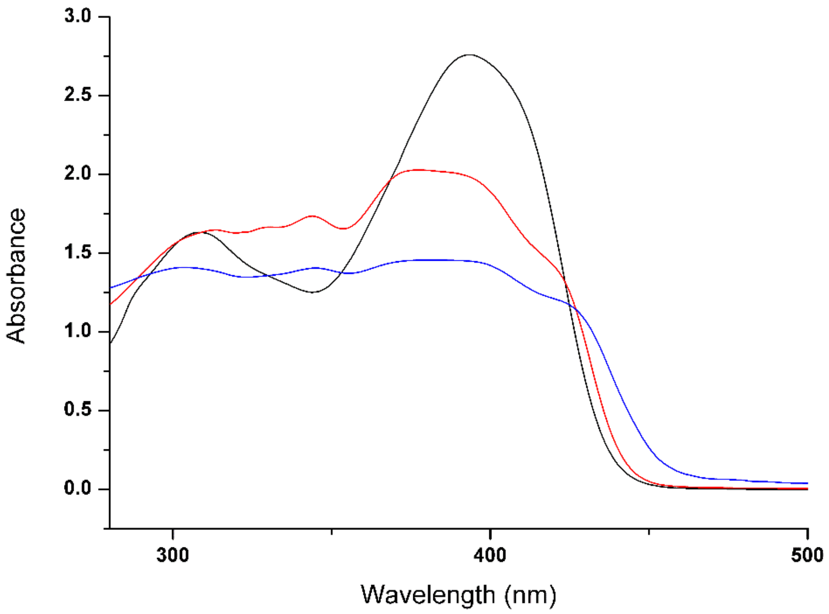

Figure S3. UV-vis absorbance spectra of TSADA in THF solution (black line), in benzene solution after heating and subsequent cooling (red line) and supramolecular thin film (blue line).

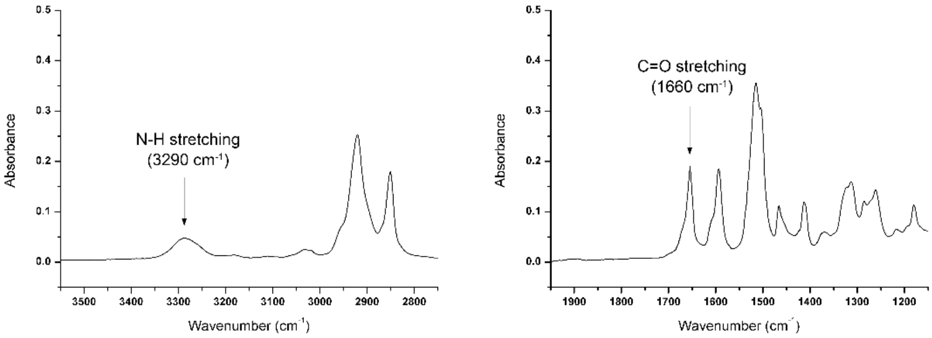

Figure S4. ATR-IR spectra of the one-dimensional self-assembled structure of TSADA in benzene.

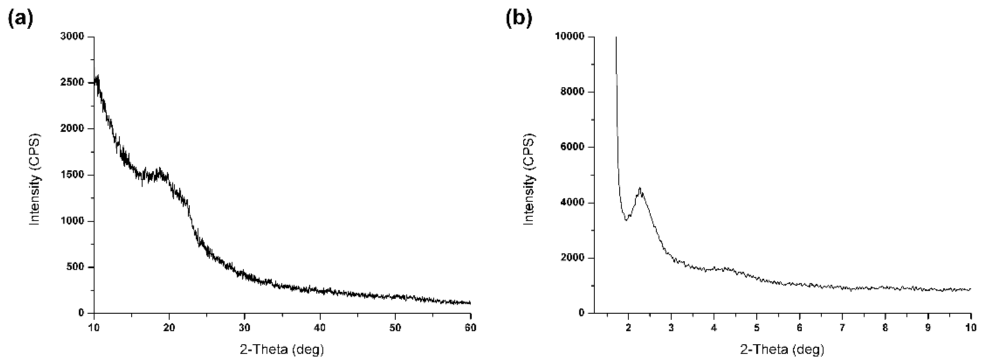

Figure S5. XRD patterns of supramolecular assemblies. (a) Wide-angle and (b) small-angle XRD patterns of the supramolecular thin film composed of supramolecular assemblies of TSADA.

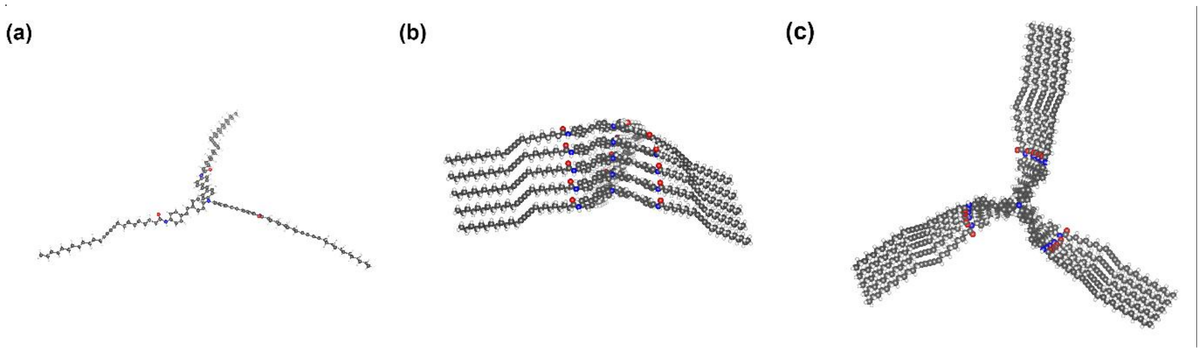

Figure S6. Estimated structure and supramolecular assembly of TSADA. Optimized structures were calculated by density-functional-tight-binding method with DFT-D3 and short-range hydrogen-hydrogen repulsion term by DFTB+. Images of (a) the structure of TSADA and pentamer stacks at (b) side view and (c) top view.

As shown in Figure S6a, the non-planar structure with tilted tris(stilbene)amine core was obtained. Consequently, the rotational direction of staggered packing was affected by the direction of tilt (Figure S6b-c). As a result, the intermolecular distance between central nitrogen atoms, the com-com (centre-of-mass) distance, was predicted as 4.02 Å, which is in agreement with the XRD result. The intermolecular distances between atoms at same position of stacked TSADA moieties vary from 4.02 Å to 4.57 Å and it also matches with the XRD observations. The length of side arm was predicted as 3.87 nm, which corresponds to the XRD peak in the small-angle regime. The rotation angle was 5.67°, which was determined by measuring dihedral angles of O-N•••N-O between two monomers. The optimized stacks exhibited that two neighboring monomers have amide hydrogen bonding with a distance of about 1.98 Å. In addition, the pentamer stacks also revealed π-π interaction with a distance of about 4.09 Å.

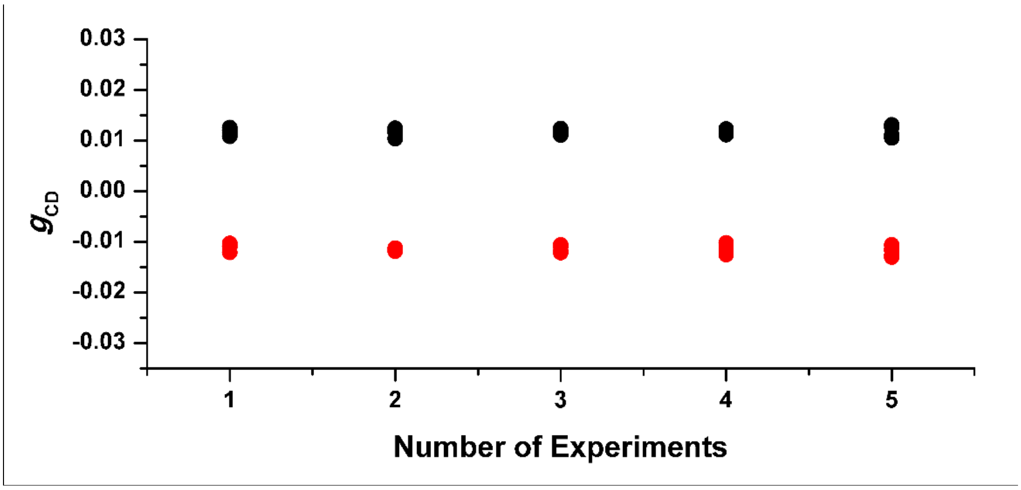

Figure S7. Repetition of generating homochiral supramolecular structure. The g_{CD} values from the samples prepared by independent experiments with irradiation of *l*-CPL (black dots) or *r*-CPL (red dots). The g_{CD} values were recorded at 431 nm and the measurements were performed at the center and four edges of the thin film for each experiment.

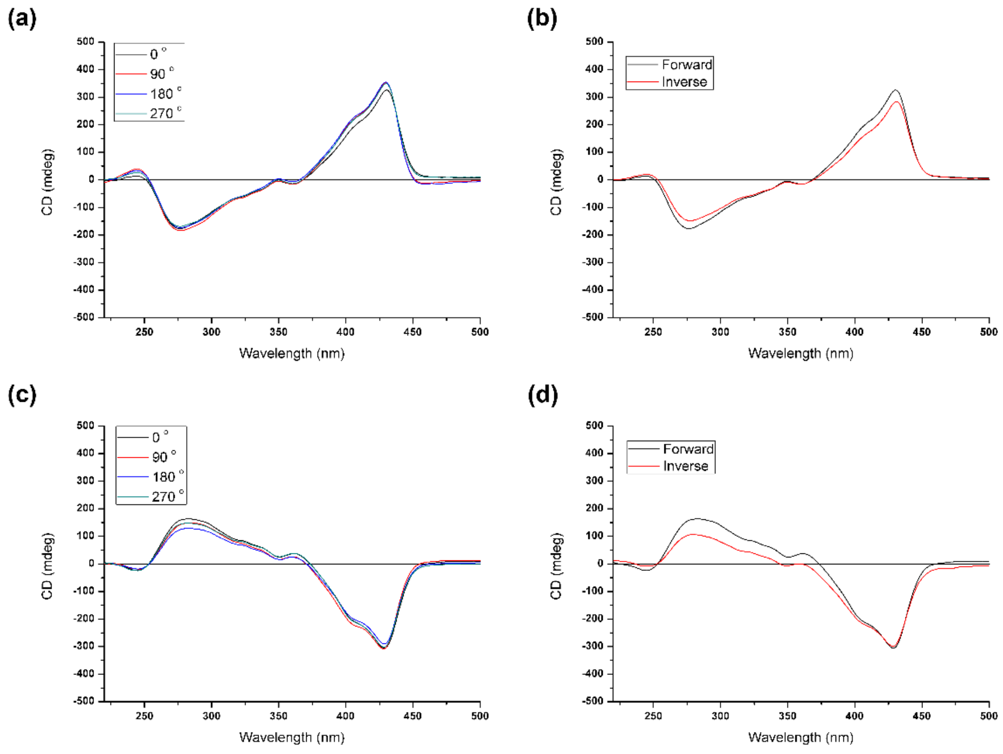

Figure S8. Rotation and inversion of homochiral supramolecular thin film. CD spectra of homochiral supramolecular thin film prepared by irradiation of (a, b) *l*-CPL and (c, d) *r*-CPL with (a, c) rotation about the normal of the surface of the thin film and (b, d) inversion of the thin film.

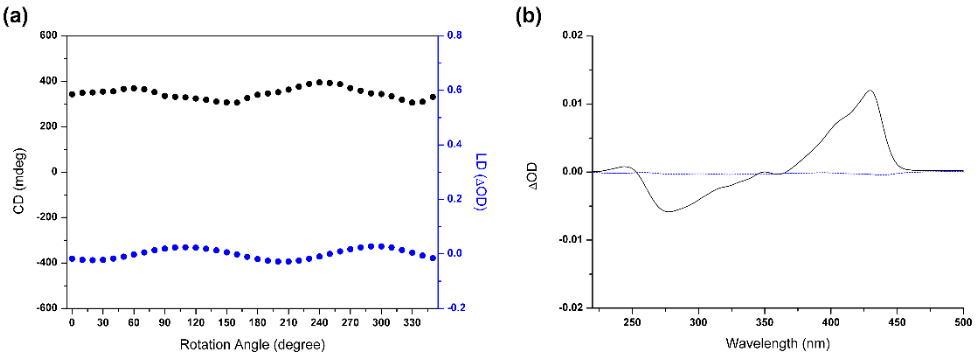

Figure S9. Estimation of the contribution of LD to CD. (a) 36 CD (black dots) and LD (blue dots) signals at λ = 431 nm measured by rotating the films about the normal of the film surface. (b) The mean spectrum of 36 CD spectra with the conversion of unit from mdeg to ΔOD for comparison (black line) and the mean spectrum of 36 LD spectra (blue line).

The CD signals fluctuated around 350 mdeg according to the rotation angle, suggesting that LD effect exists. The LD signals also fluctuated due to the macroscopic anisotropy. However, the sign of CD signals was maintained regardless of rotation angle, indicating that the CD activity mainly originates from the controlled helicity of the supramolecular assemblies. We also note that rotation of linear polarization components or anisotropic films in the film plane can eliminate the angle-dependent LD effect[1,2]. To estimate the contribution of the LD signal to the CD signal, CD spectra and LD spectra were averaged, respectively, and the contribution was quantified by using the following equation[3,4].

$$\textit{The contribution of LD to CD} = LD \times 0.02 \,/\, CD_{obs}$$

As a result, the contribution of the LD signal to the CD signal was estimated about 0.06 %.

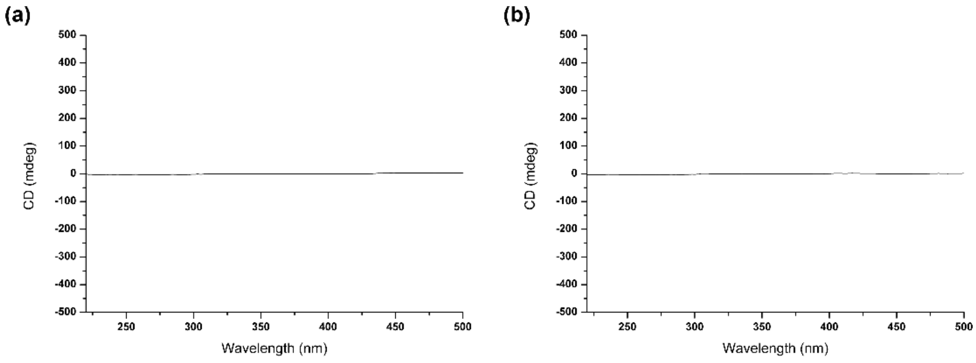

Figure S10. Racemization of the supramolecular structures under non-polarized light. CD spectra of the supramolecular thin film with control of the rotational direction of the self-assembled structures and subsequent irradiation of (a) ambient light for a day and (b) ultraviolet light for an hour.

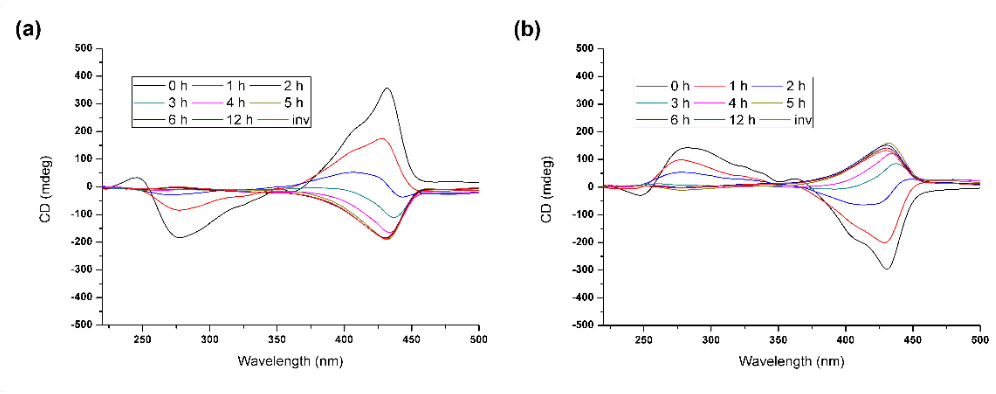

Figure S11. Time-dependent CD spectral changes of the homochiral supramolecular thin film during irradiation of CPL with opposite rotational direction. (a) Irradiation of *r*-CPL to homochiral supramolecular thin film prepared by *l*-CPL. (b) Irradiation of *l*-CPL to homochiral supramolecular thin film prepared by *r*-CPL.

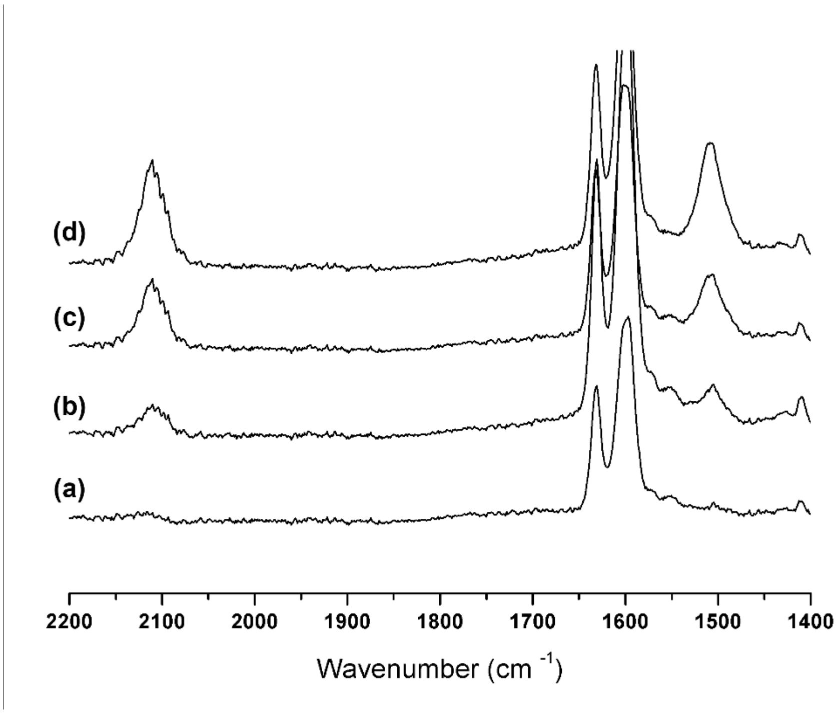

Figure S12. Locking of the self-assembled structures by photopolymerization. FT-Raman spectra of the aggregates of TSADA prepared (a) without heating and cooling process, (b) through heating and subsequent cooling without irradiation of light, (c) by heating and cooling process and subsequent irradiation of visible light and (d) by heating and cooling process and subsequent irradiation of ultraviolet light.

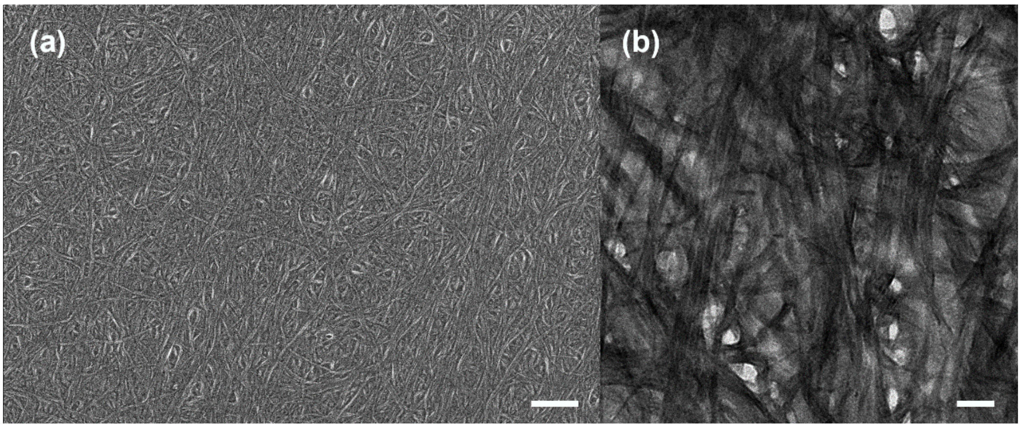

Figure S13. Microscopic images of the polymerized thin film of TSADA by irradiation of ultraviolet light (a) FE-SEM image of the polymerized thin film. Scale bar, 1 μm. (b) TEM image of the polymerized thin film. Scale bar, 200 nm.

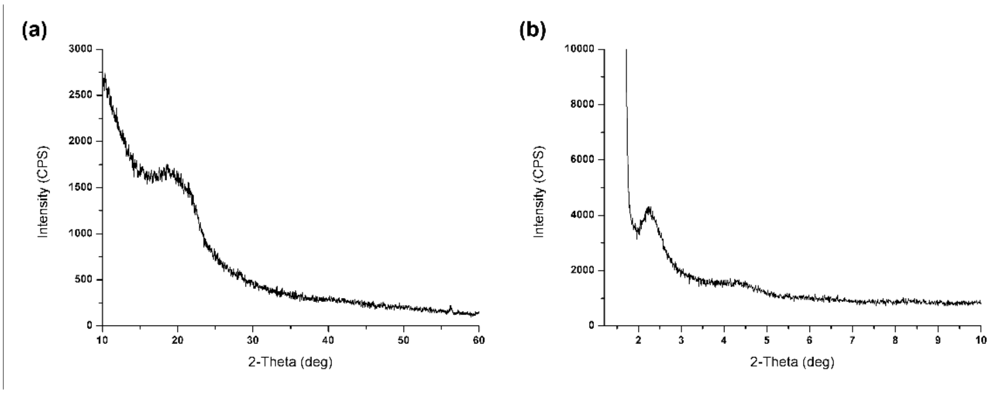

Figure S14. XRD patterns of polymerized structures. (a) Wide-angle and (b) small-angle XRD patterns of the polymerized thin film of TSADA after irradiation of ultraviolet light.

Reference

1. Narushima, T.; Okamoto, H. Circular Dichroism Microscopy Free from Commingling Linear Dichroism via Discretely Modulated Circular Polarization. *Sci. Rep.* **2016**, *6*, 35731.

2. Huang, C.-H.; Hsu, H.-S.; Sun, S.-J.; Chang, Y.-Y.; Misiuna, P.; Baczewski, L.T. Extraction of magnetic circular dichroism effects from blended mixture of magnetic linear dichroism signals in the cobalt/Scotch tape system. *Sci. Rep.* **2019**, *9*, 17192.

3. Ohira, A.; Okoshi, K.; Fujiki, M.; Kunitake, M.; Naito, M.; Hagihara, T. Versatile helical polymer films: Chiroptical inversion switching and memory with re-writable (RW) and write-once read-many (WORM) modes. *Adv. Mater.* **2004**, *16*, 1645–1650.

4. Sang, Y.; Yang, D.; Duan, P.; Liu, M. Towards homochiral supramolecular entities from achiral molecules by vortex mixing-accompanied self-assembly. *Chem. Sci.* **2019**, *10*, 2718–2724.
